# Host-encoded, cell surface-associated exopolysaccharide required for adsorption and infection by lactococcal P335 phage subtypes

**DOI:** 10.3389/fmicb.2022.971166

**Published:** 2022-10-04

**Authors:** Anne M. Millen, Dennis A. Romero, Philippe Horvath, Damian Magill, Laura Simdon

**Affiliations:** ^1^Health and Biosciences, IFF, Madison, WI, United States; ^2^Health and Biosciences, IFF, Dangé-Saint-Romain, France

**Keywords:** *Lactococcus*, P335-group bacteriophage, exopolysaccharide, capsular, receptor, plasmid, *Streptococcus thermophilus*, dairy

## Abstract

*Lactococcus lactis* and *Lactococcus cremoris* compose commercial starter cultures widely used for industrial dairy fermentations. Some lactococcal strains may produce exopolysaccharides (EPS), which have technological applications, including texture production and phage resistance. Two distinct gene clusters associated with EPS production, designated 6073-like and 7127-like, were identified on plasmids in lactococcal strains. Infectivity of two subsets of P335 group phages, distinguished based on their single-component baseplate/receptor-binding protein nucleotide sequences, was correlated to the presence of a host-encoded 6073-like or 7127-like *eps* gene cluster. Furthermore, phages belonging to these subsets differentially adsorbed to lactococcal strains harboring the respective *eps* gene cluster. Loss of the respective EPS-encoding plasmid from a fully phage-sensitive strain resulted in loss of phage adsorption and resistance to the phage. Transmission electron microscopy (TEM) showed that the EPS produced by strains encoding the 6073-like or 7127-like *eps* gene clusters are cell-surface associated, which, coupled with phage plaquing and adsorption data, shows that specific capsular EPS are involved in host recognition by certain P335 phage subgroups. To our knowledge, this is the first description of the involvement of EPS produced *via* the Wzx/Wzy-dependent pathway in phage sensitivity of *L. lactis* or *L. cremoris*. This study also shows strains that do not appear to be phage-related based on plaque formation may still be related by phage adsorption and indicates that optimal formulation of phage-robust cultures should take into account the EPS type of individual strains.

## Introduction

*Lactococcus lactis* and *Lactococcus cremoris* are lactic acid bacteria used as starter cultures for dairy fermentations. Long-term utility of highly specialized commercial strains is limited by sensitivity to bacteriophages (phages) commonly found in the fermentation environment. This can result in significant economic loss due to end-product quality issues and failed manufacturing processes. Therefore, significant research has gone into lactococcal phage-host relationships, revealing an extensive repertoire of defense systems against phages (Samson and Moineau, 2013; Mahony et al., 2014; Romero et al., 2020).

Lactococcal phages are currently classified into 10 groups, with members of the c2 (*Ceduovirus* genus), 936 (*Skunavirus* genus), and P335 groups being the most commonly found in the dairy industry (Deveau et al., 2006; Oliveira et al., 2018). To infect, phages must first recognize and bind to a receptor on the host cell. For the c2 group, the initial binding is believed to occur reversibly to a saccharidic cell wall component, then irreversibly to membrane proteins Pip or YjaE for infection (Millen and Romero, 2016). Members of other phage groups, including 936 and P335, have been shown to bind to cell wall polysaccharides (CWPS), encoded by the chromosomal *rgp* operon, that decorate the cell wall with a so-called pellicle (Ainsworth et al., 2014; Mahony et al., 2015; Romero et al., 2020). This binding is referred to as phage adsorption and is mediated by the phage adhesion modules (Mahony et al., 2017a; Goulet et al., 2020).

Many bacteria, including lactococci, produce exopolysaccharides (EPS), distinct from CWPS, *via* the Wzx/Wzy-dependent pathway (Islam and Lam, 2014). These EPS may be excreted into the growth media or remain capsular in nature (CPS); the latter is characterized as tightly associated with the cell surface (Broadbent et al., 2003). In lactic acid bacteria, the genes associated with EPS production *via* the Wzx/Wzy-dependent pathway are generally found in a cluster with a modular organization (Zeidan et al., 2017). While a high level of gene diversity is found among lactococcal *eps* clusters, *eps* genes can typically be categorized according to function (EPS assembly, EPS modulation, and glycosyltransferase; Zeidan et al., 2017; Poulsen et al., 2019). The EPS structures are also highly diverse, owing to differences in factors such as the sugar building blocks, branching, glycosidic linkages, and non-sugar decorations (Zeidan et al., 2017). This genetic and structural diversity explains the wide array of physical properties and biological functions ascribed to the EPS (Zeidan et al., 2017).

Some EPS-producing strains have commercial applications due to desirable properties that they impart in fermented dairy products, such as improved texture and moisture retention (Nachtigall et al., 2020; Korcz and Varga, 2021; Prete et al., 2021). Plasmid-encoded EPS have been described to provide phage resistance in lactococci by reducing the adsorption of phages to the cell (Lucey et al., 1992; Forde and Fitzgerald, 1999). EPS production does not universally provide protection against all phages, however, and examples of phages infecting EPS-producing lactococcal strains have been reported (Deveau et al., 2002). Additionally, EPS and CPS have been associated with phage sensitivity in another lactic acid bacterium, *Streptococcus thermophilus,* where phage-resistant mutants were found to have lost EPS production or to have developed mutations in *eps* genes (Rodríguez et al., 2008; Mills et al., 2010; Szymczak et al., 2018). Furthermore, Szymczak et al. (2018) demonstrated that cell wall-associated EPS was the likely receptor of streptococcal 987-group phages, which encode a baseplate and are genetically related to lactococcal P335 phages.

In this study, we show that two lactococcal *eps* gene clusters, designated 6073-like and 7127-like, are associated with sensitivity to specific P335 group phages. We identify two subgroups of P335 phages [distinguishable by the baseplate/receptor-binding protein (Bpp/RBP)] that adsorb to strains encoding the 6073-like or 7127-like *eps* gene clusters. Adsorption did not necessarily result in a successful infection (as measured by plaque formation in standard plate assays), but we show that loss of the plasmid encoding the *eps* gene cluster from a fully sensitive host resulted in full phage resistance. Transmission electron micrographs (TEMs) of isogenic EPS^+^/EPS^−^ strains found both EPS to be capsular in nature, consistent with their putative function as phage receptors.

## Materials and methods

### Bacterial strains and phages

Bacterial strains and phages are listed in Table 1. Lactococcal strains were grown at 30°C in sterile 11% w/v nonfat dry milk (NFDM) or in M17 broth (BD Difco) supplemented with 0.5% lactose or glucose. Note that a naturally derived *Ceduovirus* resistant *pip* mutant (6073A) was used interchangeably with DGCC6073 as a P335 phage host. When required, antibiotics were added to the media as follows: erythromycin (Em; 5 μg/ml), spectinomycin (Sp; 300 μg/ml), and streptomycin (Sm; 1,000 μg/ml). Streptococcal strains were grown at 40°C in sterile 11% w/v NFDM or in M17 broth supplemented with 0.5% sucrose.

**Table 1:** Strains, bacteriophages, and plasmids.

**Biological material**	**Relevant characteristics**	**Reference**
**Bacteria**		
*Lactococcus cremoris*		
DGCC6073	Starter strain, pEPS6073 native host	IFF Collection
6073A	Starter strain, *pip* mutant of DGCC6073, pEPS6073 native host	IFF Collection
6073AΔEPS	Phage-resistant derivative of 6073A, pEPS6073^−^	This study
6073-pG9::IS*S1*	pGhost9::IS*S1* transformant of DGCC6073, pEPS6073^+^	This study
LM2345	plasmid-free, Sp^R^	Anderson and McKay, 1984
2345-EPS	LM2345 transconjugant, pEPS6073^+^	This study
*Lactococcus lactis*		
DGCC7127	Starter strain, pEPS7127 native host	IFF Collection
7127∆EPS	pGhost9::IS*S1* transformant of DGCC7127 that was subsequently cured of pGhost9::IS*S1*, pEPS7127^−^, phage-resistant	This study
DGCC7158	Starter strain, pEPS7158 native host	IFF Collection
DGCC7204	Starter strain	IFF Collection
1403S	Spontaneous Sm^R^ derivative of IL1403	Millen et al., 2012
1403S-EPS	1403S transconjugant, pEPS6073^+^	This study
*Streptococcus thermophilus*		
DGCC7856	Starter strain, 7127-like EPS homolog	IFF Collection
7856ΔEPS	DGCC7856 EPS mutant	This study
7856Rev	DGCC7856 EPS revertant	This study
DGCC12520	Starter strain, 6073-like EPS homolog	IFF Collection
12520ΔEPS	DGCC12520 EPS mutant	This study
12520Rev	DGCC12520 EPS revertant	This study (accession number OP323076)
Bacteriophages		
D4840	P335 group, host DGCC6073	IFF Collection (accession number OP323077)
D6890	P335 group, host DGCC6073	IFF Collection (accession number OP323071)
D2544	P335 group, host DGCC7158	IFF Collection (accession number OP323072)
D2950	P335 group, host DGCC7158	IFF Collection (accession number OP323073)
D3906	P335 group, host DGCC7158	IFF Collection (accession number OP323074)
D4044	P335 group, host DGCC7204	IFF Collection (accession number OP323075)
D4351	P335 group, host DGCC7127	IFF Collection
Plasmids		
pGhost9::IS*S1*	Em^R^, Temperature sensitive vector, insertion sequence IS*S1*	Maguin et al., 1996
pEPS7127	7127-like EPS-associated genes	This study
pEPS6073	6073-like EPS-associated genes	This study
pEPS7158	6073-like EPS-associated genes (EpsM variant)	This study

Phages were isolated from industrial whey samples from Europe or Africa between 1999 and 2014. Preparation of phage lysates was performed as previously described (Terzaghi and Sandine, 1975). High titer lysates were passed through a 0.45 μm filter and stored at 4°C. Spot titer assays and plaque assays were performed as previously described (Terzaghi and Sandine, 1975) on MRS medium (BD Difco). Phage adsorption tests were performed as previously described (Sanders and Klaenhammer, 1980) on MRS medium.

### Phage challenge

Single-step phage challenges were performed by exposing log-phase 6073A and DGCC7127 to phage D4840 or D4351, respectively, at a multiplicity of infection (MOI; number of phages relative to the number of cells) between 1 and 10 and then plating onto MRS in soft agar overlay. Single, phage-resistant colonies arising from the 6073A challenge were selected; however, DGCC7127 challenge plates all grew poor bacterial lawns rather than the expected clearing and formation of resistant colonies. Therefore, regions of the lawns were streaked onto MRS plates containing a high titer of D4351 in soft agar overlay to isolate single phage-resistant colonies.

### Electroporation and conjugation

Electroporation of lactococci was performed as previously described (Holo and Nes, 1989). Solid surface conjugal mating was performed as previously described (McKay et al., 1980), except that M17 medium containing 0.5% glucose and 0.5% lactose was substituted for milk agar plates. To facilitate tracking of conjugal transfer, pGhost9::IS*S1* was first introduced into prospective donors. IS*S1* promotes recombination with a conjugative element enabling tracking *via* erythromycin resistance encoded on pGhost9. Selection for transconjugants was performed on M17 medium supplemented with the respective carbohydrate and antibiotics. Putative transconjugants, identified by erythromycin resistance, were then predicted to be positive for acquisition of pEPS6073 based on testing positive in a PCR reaction targeting the *epsC* gene (pEPS6073_03). Transconjugants were obtained at efficiencies of >9 × 10^−9^ per exit recipient. pGhost9::IS*S1* was cured from transconjugants by growth in milk at 37°C in the absence of erythromycin.

### PCR, DNA preparation, sequencing, and *in silico* analysis

PCRs were performed with GoTaq® Colorless Master Mix (Promega Corp., United States) or Phusion HF Master Mix (New England Biolabs, United States) according to the manufacturer’s instructions. Primers were synthesized by Integrated DNA Technologies (United States). Primer sequences are listed in Supplementary Table 1. Plasmid DNA was prepared as previously described (Anderson and McKay, 1983). Phage DNA was isolated from high titer phage lysate using Invitrogen PureLink Viral RNA/DNA Mini kit (Life Technologies, United States) according to the manufacturer’s instructions. Phage DNA was sequenced using Illumina technology (Illumina, United States). Genomic DNA was isolated from lactococci for whole-genome sequencing using the EpiCentre MasterPure Complete DNA and RNA Purification kit (EpiCentre, United States). Illumina and PacBio or Nanopore sequencing reads were used in hybrid assemblies completed with the Unicycler pipeline (Wick et al., 2017), which includes Miniasm and Racon pipelines and Pilon polishing. DNA sequences were annotated using PATRIC RASTtk-enabled Genome Annotation Service (Brettin et al., 2015) and/or Prokka (Seemann, 2014) prior to manual curation. In certain cases, HHpred was used for protein homology detection (Söding et al., 2005). Genomes were analyzed using Geneious Prime 2021.0.3.^1^ Genetic organization depictions were generated with the EasyFig 2.2.5 software (Sullivan et al., 2011).

### Natural competence and selection of transformants

Transformation of *S. thermophilus* was performed using natural competence (Fontaine et al., 2010). Gblocks, which served as templates for the transformation amplicons, were designed to introduce mutations in the respective *eps* gene: T > G at position 599 in the DGCC12520 6073-like signature gene (named DGCC12520_eps21) and AA > TG at positions 784–785 in the DGCC7856 7127-like signature gene (named DGCC7856_eps20). 1 ml of chemically defined medium (Poolman and Konings, 1988; Juillard et al., 1998) was inoculated at 2% with frozen cell stock and incubated overnight at 37°C. This overnight culture was used to inoculate 1 ml of sterile 11% (w/v) NFDM at 5%. Inoculated milk was incubated at 37°C for 75 min. Following incubation, ComS inducer peptide (peptide sequence N-terminal to C-terminal: LPYFAGCL; final concentration = 1 μM) was added to the inoculated milk. 300 μl of this culture was mixed with 1–2 μg of respective PCR product (Supplementary Table 2) and then incubated at 37°C for 3 h followed by spread plating on FSDA II medium (Sandine, 1985), with the trimagnesium phosphate substituted with β-glycerophosphate (19 g per 100 ml deionized water). Plates were incubated at 37°C anaerobically overnight. Resulting single isolated colonies were tested for mutations with respective primers (Supplementary Tables 1, 2). Positive samples were purified by plating on FSDA II and selecting single colonies. Colonies were inoculated into sterile 11% (w/v) NFDM which was then incubated at 40°C overnight.

### Transmission electron microscopy

Transmission electron microscopy (TEM) analyses were performed by the Electron Microscopy Lab VA-MD College of Veterinary Medicine (Blacksburg, VA, United States). 1 ml of overnight bacterial cultures was centrifuged. Pellets were resuspended in 1 ml fixative (3% glutaraldehyde in 0.1 M sodium cacodylate buffer pH 7.4), followed by fixation in osmium tetroxide (2%), and serial dehydration in ethanol (15, 25, 75, 95, and 100%), before samples were embedded in Poly/Bed812 resin. 100 nm thick sections were collected on copper grids and stained with uranyl acetate (3%) and lead citrate (Reynolds solution). TEM images were acquired using a JEM-JEOL-1400 (80 kV) system.

## Results

### Bacteriophage insensitivity correlated to plasmids that encode *eps* gene clusters

Bacteriophage insensitive mutants (BIMs) of 6073A and DGCC7127 were generated against homologous P335 phages by high titer phage challenge. Four 6073A survivors were tested following challenge with phage D4840. All tested fully resistant to homologous phages D4840 and D6890, which were independently isolated 6 years apart (Table 2). Plasmid profiling found differences in plasmid content among 6073A BIMs (data not shown); notably, all BIMs had lost a large plasmid. Phage-resistant variants of parental strain DGCC6073 were also generated without the use of phage selection. Upon electroporation with pGhost9::IS*S1*, five out of nine DGCC6073 transformants were found to have lost a plasmid equivalent in size to the plasmid lost in the BIMs and were resistant to phages D4840 and D6890. Comparing the genome sequence of DGCC6073 with that of a BIM isolated from the phage challenge, designated 6073A∆EPS, found that the BIM had lost a 34.3 kb plasmid encoding genes associated with EPS production *via* the Wzx/Wzy-dependent pathway; we designated this plasmid as pEPS6073.

**Table 2:** Phage plaque assays.

		**Efficiency of plaquing (EOP)**				
**6073-like strains**	**EPS**					
		**D4840**	**D6890**	**D2544**	**D2950**	**D3906**
DGCC6073	+	1	1	NT	NT	NT
6073A∆EPS	**−**	<4 × 10^−9^	<4.3 × 10^−9^	NT	NT	NT
2345-EPS	+	<4 × 10^−7^^*^	2.1 ± 1.04, turbid	<1.6 × 10^−8^^*^	<8.9 × 10^−10^	<1.6 × 10^−7^^*^
LM2345	**−**	<4 × 10^−9^	<4.3 × 10^−9^	<1.6 × 10^−9^	<8.9 × 10^−10^	<1.6 × 10^−9^
1403S-EPS	+	<4 × 10^−9^	<4.3 × 10^−9^	1.7 × 10^−8^ ± 1.5 × 10^−9^	<8.9 × 10^−10^	<1.6 × 10^−9^
1403S	**−**	<4 × 10^−9^	<4.3 × 10^−9^	<1.6 × 10^−9^	<8.9 × 10^−10^	<1.6 × 10^−9^
DGCC7158	+	NT	NT	1	1	1
**7127-like strains**						
		**D4351**	**D4044**			
DGCC7127	+	1	<8.5 × 10^−10^			
7127∆EPS	**−**	<3.0 × 10^−10^	<8.5 × 10^−10^			
DGCC7204	+	<3.0 × 10^−10^	1			

When applicable, EOP is the average of three independent trials ± sample standard deviation. When no plaques were visible, EOP is < the highest value of three independent trials. * no plaquing; poor bacterial lawn/clear when exposed to high levels of phage, but phage did not titer out to single plaques. In this case EOP is based on the highest dilution of phage where bacterial lawn inhibition was observed.

For DGCC7127, six survivors from challenge with phage D4351 were tested, and all were fully resistant to phage D4351 (Table 2). Plasmid profiling did not discern a difference in plasmid content in DGCC7127 BIMs; however, suspecting results similar to 6073A, the survivors were tested for the presence of *eps* genes. All BIMs tested negative in a PCR reaction with primers EpsC-F and EpsC-R, designed to amplify conserved lactococcal EPS-associated gene *epsC,* while parent DGCC7127 was positive (data not shown). Phage-resistant variants of DGCC7127 were also generated without phage selection. Upon electroporation with pGhost9::IS*S1*, three out of six DGCC7127 transformants tested were found to be phage-resistant, and phage-resistant transformants were found to be negative in a PCR with EpsC-F and EpsC-R primers. The genome sequence of a representative transformant cured of pGhost9:IS*S1* (designated 7127∆EPS) revealed the loss of a 38.3 kb plasmid, designated pEPS7127, that encodes a set of genes associated with EPS production distinct from the set encoded by pEPS6073. Detection of pEPS7127 in standard gel electrophoresis profiles is occluded by the presence of another similarly-sized plasmid of 37.3 kb. As no other known gene associated with phage resistance was found on pEPS6073 or pEPS7127, and the loss of the plasmids from their respective hosts represented the principal genetic difference between BIM and parent, we chose to investigate the involvement of EPS in phage-host interaction further.

### Characterization of plasmid-encoded *eps* gene clusters

**Figure 1:**
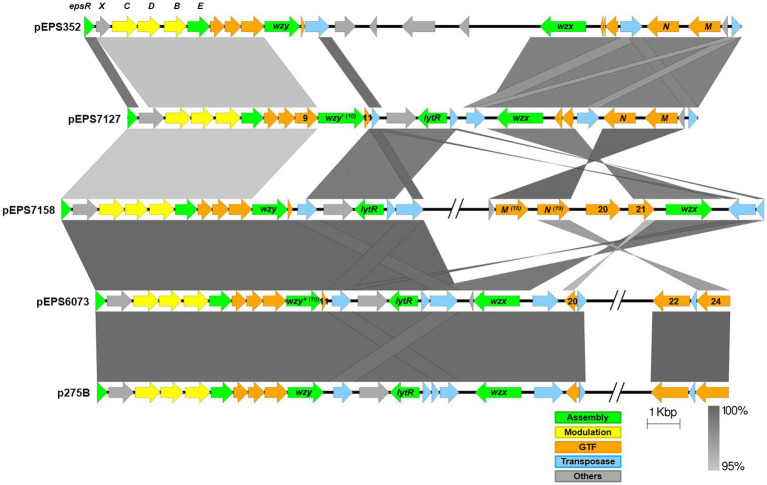
Lactococcal *eps* gene clusters. Comparison of IFF lactococcal *eps* gene clusters associated with P335 phage sensitivity and selected publicly available lactococcal *eps* gene clusters. 6073-like EPS is represented by pEPS6073 and pEPS7158 (EpsM variant). 7127-like EPS is represented by pEPS7127. Numbers appearing over gene depictions correspond to locus tags as annotated in sequences submitted to GenBank. Genes are colored based on their putative function, including EPS assembly, EPS modulation, glycosyltransferase, transposase, or others. The putative polymerase (*wzy*), flippase (*wzx*), and attachment (*lytR*) genes are annotated as such. *indicates designated signature genes. Gray bars are used to indicate sequence identity.

The *eps* gene clusters encoded on pEPS6073 and pEPS7127, designated as 6073-like and 7127-like, respectively, are depicted in Figure 1. pEPS6073 harbors 17 coding sequences (CDS) associated with exopolysaccharide production, which are highly conserved with those found on lactococcal plasmid p275B (accession number CP016700). A closely related variant of the 6073-like *eps* gene cluster, which we designated EpsM variant, was identified in several IFF collection strains and is described here as it resides on plasmid pEPS7158 in *L. lactis* DGCC7158. The EpsM variant can be distinguished from the typical 6073-like cluster by differences in a set of two glycosyltransferase (GTF) genes found outside of the contiguous *eps* operon. pEPS6073 contains GTF genes pEPS6073_22 and pEPS6073_24, whereas pEPS7158 contains GTF genes annotated as *epsM* (pEPS7158_18) and *epsN* (pEPS7158_19), whose products share amino acid identity to the respective proteins encoded on lactococcal plasmid pEPS352 (Knoshaug et al., 2007). While pEPS7158 EpsN shares about 92% amino acid identity with the product of pEPS6073_24, EpsM has no significant amino acid identity to the product of pEPS6073_22. Additionally, a partial putative polysaccharide polymerase gene resides upstream of *epsM* in pEPS7158 that is not present in pEPS6073 and is unannotated in pEPS352. Lastly, pEPS7158 contains two additional GTF genes (pEPS7158_20 and pEPS7158_21) upstream of the flippase (*wzx*). The 3′ end of pEPS7158_21 shares 96% nucleotide identity with pEPS6073_20. Of the *eps* genes specific to pEPS7158, only *epsM and epsN* are consistently found in all IFF collection strains that contain the 6073-like EpsM variant EPS (data not shown). The difference in GTF content between the 6073-like strain and its variant may significantly affect the composition of the EPS. GTFs are responsible for linking the sugars of the EPS, and each GTF may have different substrate specificity (van Kranenburg et al., 1999).

pEPS7127 bears 19 CDS associated with EPS production. Sixteen CDS are highly conserved with those found in the 6073-like clusters, with their deduced protein sequences sharing >94% amino acid identity. The pEPS7127_09 GTF is less conserved, sharing 70.6% amino acid identity with its homolog from the 6073-like clusters, and two CDS (pEPS7127_10 and pEPS7127_11) are unique to the 7127-like locus. Of these unique CDS, pEPS7127_11 appears to be truncated by a mobile element based on its position and its closest BLAST matches. Therefore, pEPS7127_10 (putative Wzy) was designated as the signature gene for the 7127-like *eps* cluster. pEPS6073_10 and pEPS6073_11 are the only CDS found in both 6073-like *eps* clusters that are not also found in the 7127-like cluster. Due to the small size of pEPS6073_11 (144 bp) and its location next to a mobile element, pEPS6073_10 (putative Wzy) was designated the signature gene for the 6073-like *eps* gene cluster.

### Electron microscopy

**Figure 2:**
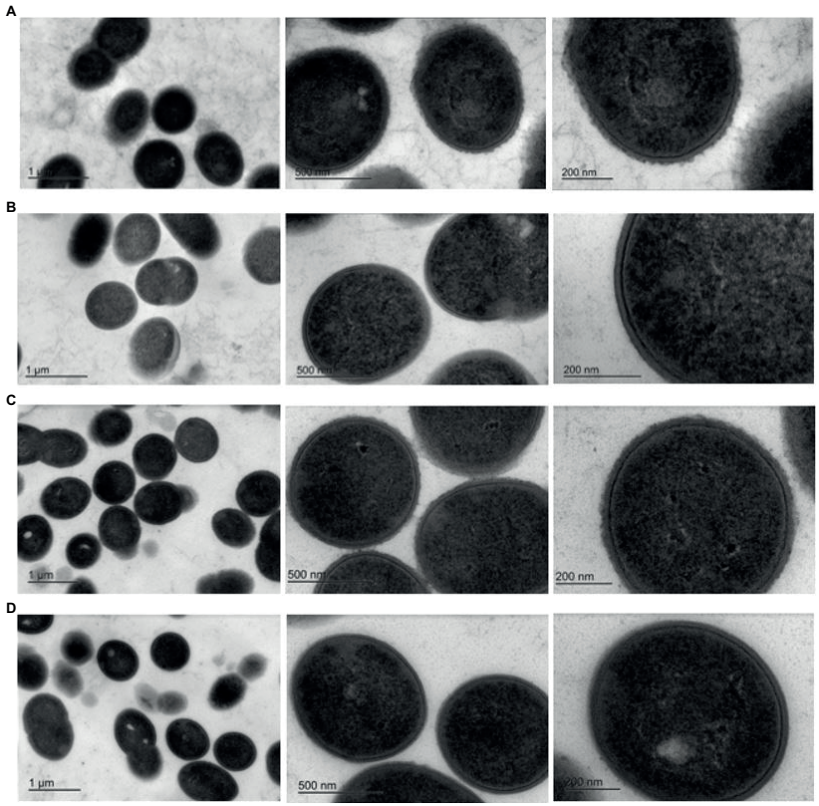
Transmission electron micrographs at three different magnifications. Row **(A)**. 6073-like EPS^+^ strain DGCC6073; Row **(B)**. EPS^-^variant 6073A∆EPS; Row **(C)**. 7127-like EPS^+^ strain DGCC7127; Row **(D)**. EPS^-^variant 7127∆EPS.

Exopolysaccharides may be excreted into the growth media or remain capsular in nature (Broadbent et al., 2003). TEM was performed to determine the nature of the EPS encoded by pEPS6073 and pEPS7127. Electron micrographs of DGCC6073 and DGCC7127 showed uniform fibrillated projections extending from the cell wall that are absent from respective EPS-negative variants 6073A∆EPS and 7127∆EPS, indicating that the EPS are associated with the cell surface (Figure 2). These data, coupled with the fact that growth of DGCC6073 or DGCC7127 was not shown to substantially affect milk texture, which would be expected if EPS was excreted into the growth medium (Knoshaug et al., 2007; Korcz and Varga, 2021; Prete et al., 2021), indicate the EPS produced by DGCC6073 and DGCC7127 are likely to be capsular.

### Phage adhesion module analysis and screening

**Figure 3:**
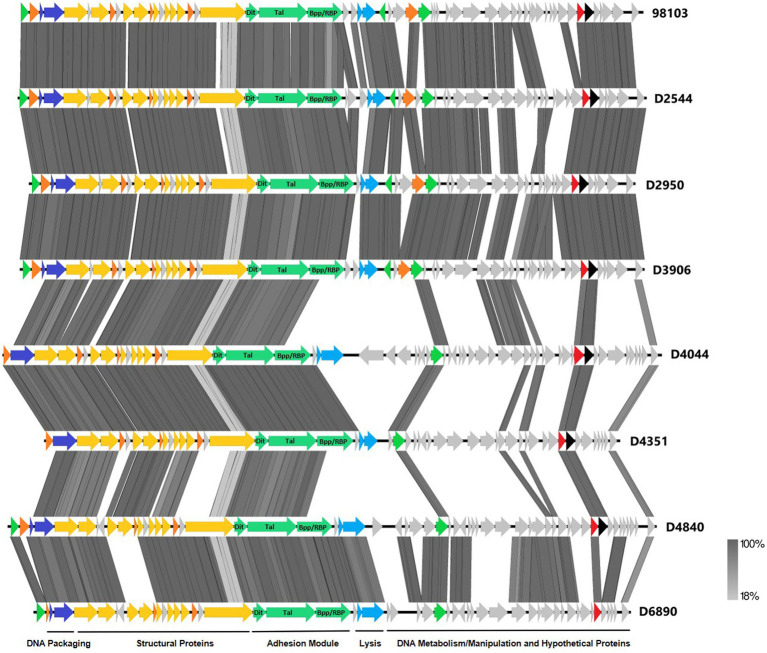
Whole-genome alignment of P335 phages highlighting the localization of the adhesion module. IFF P335 phage genomes described in this study along with phage 98103 were globally aligned. Regions of similarity exhibiting a minimum identity overlap of 100 bp are shown in varying scales of gray according to the percentage identity observed. Genes exhibiting globally related functions possess the same color scheme and are identified according to the modular descriptions provided at the bottom of the figure.

**Figure 4:**
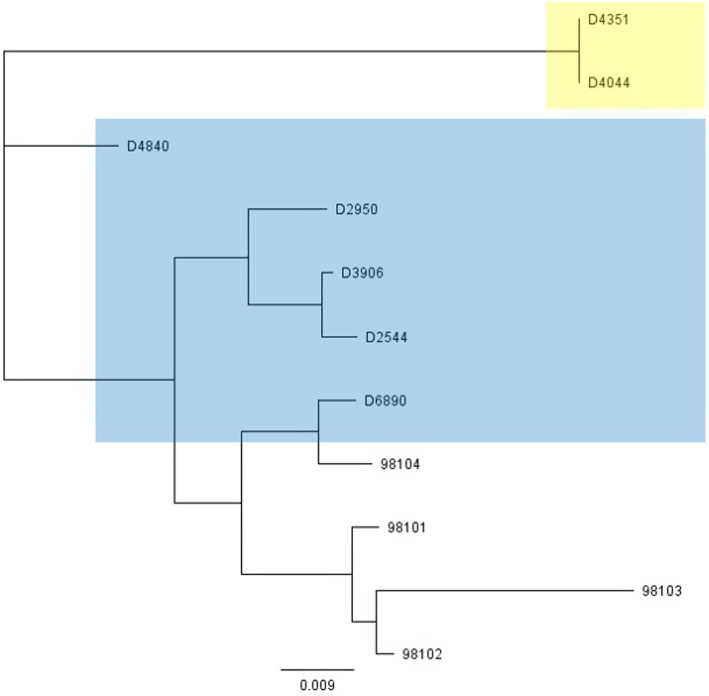
Phylogenetic tree showing nucleotide relatedness of the genes encoding the Bpp/RBPs of a subset of P335-group phages. Phages highlighted in blue infect strains encoding the 6073-like *eps* gene cluster. Phages highlighted in yellow infect strains encoding the 7127-like *eps* gene cluster. Phages that are not highlighted are public domain phages, and we have no data on their host *eps* gene content (Mahony et al., 2017b).

To further investigate why loss of pEPS6073 provides resistance to D4840 and D6890, the phage genomes were sequenced to help identify the genetic determinants associated with this phenotype. We hypothesized that the phages may be recognizing the host EPS as a receptor; therefore, we analyzed the phage adhesion modules [including distal tail protein (Dit), tail-associated lysin (Tal), and baseplate/receptor-binding protein (Bpp/RBP)], as they are responsible for attachment to the host (McCabe et al., 2015; Mahony et al., 2017a,b; Hayes et al., 2019). The Dit and Tal proteins of D4840 and D6890 were found to be conserved with those of P335 morphotype II adhesion modules (Figure 3). Both phages encode a single-component Bpp/RBP that shows conservation only with a subset of the morphotype II phages. The analysis of all publicly available phage sequences as well as internal IFF collection phages determined that the Bpp/RBPs of D4840 and D6890 are most closely related to those of P335 morphotype II phages 98101, 98102, 98103, and 98104 (Mahony et al., 2017b). This analysis also identified three additional sequenced IFF collection P335-type phages encoding Bpp/RBPs that share a high nucleotide identity with that of D4840. These phages (D2950, D2544, and D3906) all infect host DGCC7158, which encodes the 6073-like EpsM variant *eps* gene cluster. Searching for stronger identity levels over shorter sequences identified D4351 and one additional IFF collection P335 phage, D4044, as encoding Bpp/RBPs that are distinct but related to those identified in the previous analysis (Figure 4). Notably, the host strains of D4044 and D4351 (DGCC7204 and DGCC7127, respectively) both encode a 7127-like *eps* gene cluster.

### Phage sensitivity associated with pEPS6073 and pEPS7127

To further study the P335 phage-host interactions, we attempted to conjugally transfer the EPS plasmids into plasmid-free model lactococcal strains LM2345 and 1403S. A representative phage-sensitive pGhost9::IS*S1* transformant of DGCC6073 (6073-pG9::IS*S1*) was used as the pEPS6073 donor strain. Four out of 24 putative LM2345 transconjugants tested and three out of 20 putative 1403S transconjugants tested were predicted to be positive for acquisition of pEPS6073 based on testing positive in a PCR reaction targeting the *epsC* gene (pEPS6073_03). Genome sequencing of representative transconjugants 2345-EPS and 1403S-EPS confirmed the transfer of the entire pEPS6073 and confirmed the sequence integrity of the *eps* gene cluster.

To ascertain the impact of pEPS6073 on phage susceptibility, 2345-EPS and 1403S-EPS were tested in standard plaque assays with phages D4840 and D6890. These phages do not infect LM2345 and 1403S, and transconjugant 1403S-EPS did not show an increase in sensitivity to these phages; however, transconjugant 2345-EPS displayed sensitivity to both phages (Table 2). While no visible plaque formation was observed when assayed with D4840, 2345-EPS developed either no lawn or weak growth when exposed to high levels of phage (phage titer >1 × 10^8^ pfu/ml). Growth of parent LM2345 was unaffected at the same high phage levels. D6890 did form plaques on 2345-EPS at higher efficiency than on propagating host DGCC6073; however, plaques were turbid on 2345-EPS. Transconjugants were also tested against phages D2950, D2544, and D3906, all of which encode a Bpp/RBP with nucleotide identity to those of D4840 and D6890. D2950 and D3906 did not form plaques but D2544 did on 1403S-EPS. No plaque was observed on 2345-EPS with any of the three phages; however, 2345-EPS was inhibited by D2544 and D3906, similar to what was observed with phage D4840. These data confirm pEPS6073 is associated with sensitivity to certain P335 phages.

pEPS7127 could not be conjugated into the model strains. Therefore, to further understand the role of the 7217-like gene cluster in phage susceptibility, plaque assays with phages D4044 and D4351 were performed on isogenic strain pair DGCC7127/7127∆EPS as well as D4044 host DGCC7204. Results showed that each phage formed plaques only on its propagating host and confirmed that 7127∆EPS was fully resistant to D4351 (Table 2).

### 6073-like and 7127-like EPS facilitate adsorption of a respective subset of P335 phages

**Figure 5:**
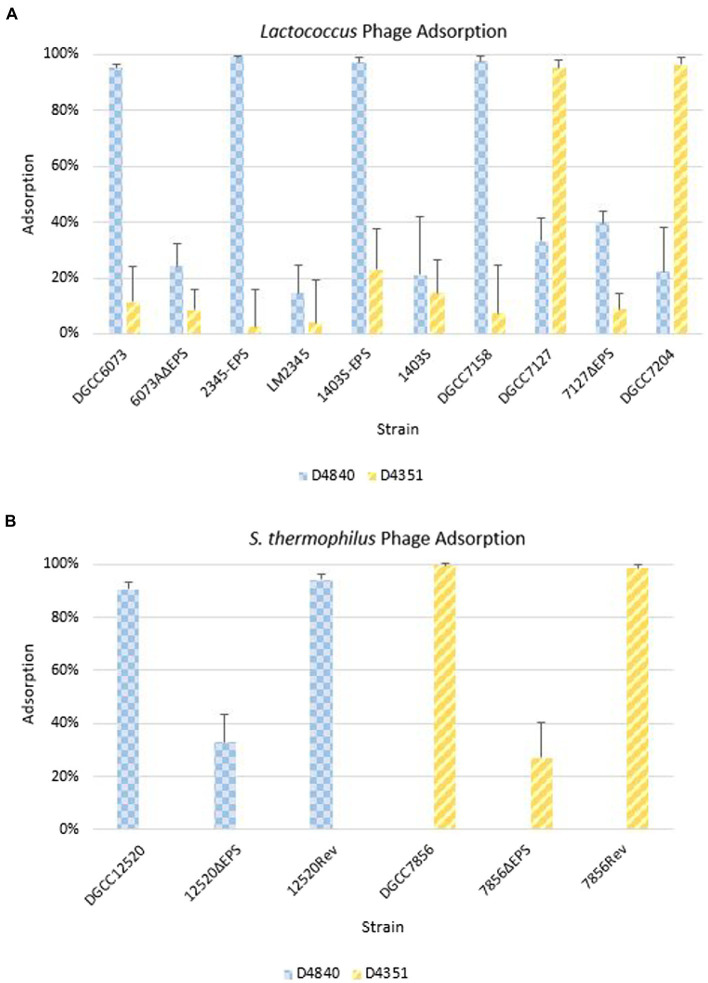
Phage adsorption on representative strains including isogenic strain pairs (+/− EPS). Average of three independent trials. Error bars = sample SD. **(A)** Adsorption on *Lactococcus* strains. **(B)** Adsorption on *Streptococcus thermophilus* strains.

Phage adsorption assays were performed to ascertain whether phages were recognizing strains containing pEPS6073, despite the lack of visible plaque formation in assays (Figure 5A). Phage D4840 adsorbed to transconjugants 2345-EPS and 1403S-EPS equivalently to homologous host DGCC6073 (>90%) as well as to EpsM variant strain DGCC7158. Accordingly, D4840 was unable to adsorb to strains in which the plasmid was absent (parental recipients LM2345 and 1403S) or lost (6073AΔEPS). This result was extended to related phages encoding the D4840 Bpp/RBP (Figure 4); namely D6890 and phages D2544, D2950, and D3906 whose homologous host DGCC7158 contains the 6073-like EpsM variant (Supplementary Figure 1).

For pEPS7127, its presence enabled high-level adsorption by homologous phage D4351, whereas loss of the plasmid (7127∆EPS) resulted in concomitant reduction of phage adsorption (Figure 5A). Likewise, non-homologous phage D4044, which encodes a Bpp/RBP with identity to D4351 (Figure 4) and whose homologous host (DGCC7204) contains a 7127-like *eps* gene cluster, adsorbed to DGCC7127 at a level equivalent to its adsorption to DGCC7204 (Supplementary Figure 1) despite not forming visible plaques on the former. Expectedly, D4351 also adsorbed to DGCC7204 at a high level (Figure 5A). Collectively, these results corroborate the involvement of the 6073-like and 7127-like EPS in the differential adsorption of a specific subgroup of P335 phages, which can be distinguished by their Bpp/RBP sequence.

### 6073-like and 7127-like *eps* gene clusters in *Streptococcus thermophilus*

P335 lactococcal phage 98103, which encodes a Bpp/RBP with nucleotide identity to phages that adsorb to strains encoding the 6073-like EPS, was reported to also adsorb to a *S. thermophilus* strain (McDonnell et al., 2016). We theorized that the *S. thermophilus* strain may produce a lactococcal 6073-like EPS, which may explain this adsorption phenotype. The designated signature genes for the 6073-like and the 7127-like lactococcal *eps* gene clusters were searched within the IFF collection of *S. thermophilus* genomes. *S. thermophilus* DGCC12520 and DGCC7856 were found to encode the 6073-like and the 7127-like signature gene, respectively.

**Figure 6:**
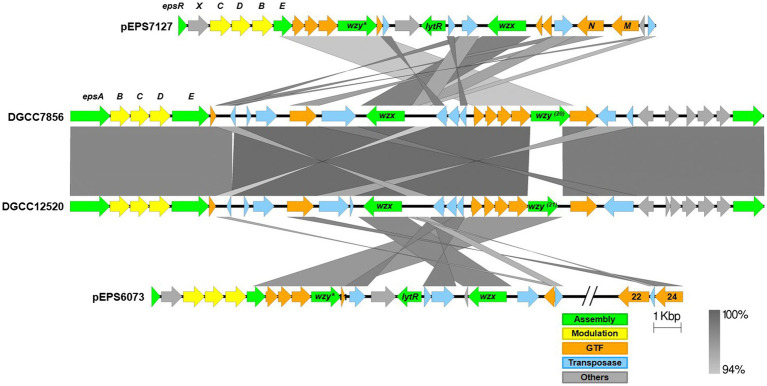
Alignment of streptococcal and lactococcal *eps* gene clusters. Comparison of lactococcal 6073-like and 7127-like *eps* gene clusters with related streptococcal clusters. Lactococcal 6073-like EPS is represented by pEPS6073, and 7127-like EPS is represented by pEPS7127. Numbers appearing over gene depictions correspond to locus tags as annotated in sequences submitted to GenBank. Genes are colored based on their putative function, including EPS assembly, EPS modulation, glycosyltransferase, transposase, or others. The putative polymerase (*wzy*), flippase (*wzx*), and attachment (*lytR*) genes are annotated as such. *indicates designated signature genes. Gray bars are used to indicate sequence identity.

Alignments of the *eps* gene clusters of *S. thermophilus* DGCC12520 and DGCC7856 with their respective lactococcal *eps* clusters can be found in Figure 6. The two *S. thermophilus* clusters are highly related, except DGCC12520 encodes the signature gene (DGCC12520_eps21) for the 6073-like EPS and DGCC7856 encodes the signature gene (DGCC7856_eps20) for the 7127-like EPS. The *eps* gene clusters are modular, with the genes located at the 5′ end, which are involved in EPS modulation and assembly, being highly conserved within each species (Zeidan et al., 2017; Poulsen et al., 2019). However, despite being functionally related, these modules (lactococcal *epsRXCDB* and streptococcal *epsABCDE*) do not share any significant identity. Downstream of this species-conserved region, the *S. thermophilus* clusters encode a set of GTFs with identity to GTFs found in the respective lactococcal clusters, and the *S. thermophilus* flippase and putative polymerases shares high identity with that of *Lactococcus*. This is likely a result of horizontal gene transfer, which has been previously described for *eps* genes (Liu et al., 2009).

The *S. thermophilus* strains were tested against a representative phage belonging to the lactococcal P335 phage subset associated with their respective EPS type; D4840 (6073-like) on DGCC12520 and D4351 (7127-like) on DGCC7856. Both phages adsorbed at 90–99% to the respective *S. thermophilus* strain (Figure 5B). To confirm adsorption was due to EPS, the relevant signature *eps* gene in each *S. thermophilus* was disrupted by introduction of a stop codon generating a truncated protein. Adsorption of lactococcal phages by representative mutants 12520ΔEPS and 7856ΔEPS was reduced to approximately 33 and 27%, respectively (Figure 5B). The mutated signature *eps* genes were then reconstituted, which restored lactococcal phage adsorption to wild-type strain levels of 90% or greater. These results are consistent with the hypothesis that the 6073-like and 7127-like EPS are involved in the differential adsorption of a specific subgroup of P335 phages.

## Discussion

Like many bacteria, lactococci may produce EPS *via* the Wzx/Wzy-dependent pathway (Islam and Lam, 2014). When present, the genetic determinants for EPS production in lactococci are often plasmid-encoded (Kelleher et al., 2019). Here, we describe lactococcal plasmid-encoded 6073-like and 7127-like *eps* gene clusters, each of which was found to be correlated to sensitivity to a subgroup of P335 phages. BIMs of *L. cremoris* 6073A and *L. lactis* DGCC7127 were found to have lost their respective EPS-encoding plasmid (pEPS6073 or pEPS7127), and model strains LM2345 and 1403S showed increased sensitivity to select P335 phages after conjugal transfer of pEPS6073. In several cases, this increased phage sensitivity was not apparent from phage plaque formation; rather, growth of the bacterial lawn was poor in the presence of high phage concentrations. It is currently unclear what causes this phenotype. It is possible that the EPS makes the cell more susceptible to the phage lysin. It could also be that there is a cost to adsorption of certain phages, as adsorption assays on isogenic 6073-like EPS^+^/EPS^−^ strains showed they adsorbed phages belonging to the respective P335 subgroup at a high level regardless of whether they showed sensitivity in a plaque assay. Likewise, adsorption assays on isogenic 7127-like EPS^+^/EPS^−^ strains found only the EPS^+^ strains adsorbed phages belonging to the respective phage subgroup. Additionally, *S. thermophilus* strains encoding homologs of the 6073-like or 7127-like EPS were able to adsorb the respective lactococcal phages. Adsorption by *S. thermophilus* was lost upon mutation of the signature *eps* gene and restored when the gene was reverted to wild type, confirming the roles of the *eps* gene clusters in phage adsorption. Overall, these data indicate that certain P335 phages use EPS as a receptor.

Chromosomally-encoded lactococcal cell wall polysaccharides (CWPS), which decorate the cell surface and are distinct from plasmid-encoded EPS described in this study, have been previously shown to play a role in phage adsorption, including phages of the P335 group (Ainsworth et al., 2014; Mahony et al., 2015; Romero et al., 2020). Variability in the *rgp* gene clusters has allowed for strains to be typed, and CWPS type has been correlated with phage sensitivity (Ainsworth et al., 2014; Murphy et al., 2016; Romero et al., 2020). We have not investigated whether EPS production has any impact on CWPS production; however, all strains in this study encode presumably functional *rgp* operons based on genetic analyses (data not shown). Additionally, certain phages belonging to the genus *Skunavirus*, which are known to use the CWPS as receptors (Ainsworth et al., 2014), infect both 6073A and 6073AΔEPS equivalently (data not shown), indicating that the receptor is unaffected by EPS production. Although both LM2345 and DGCC6073 encode C.1 CWPS, 1403S encodes type B and DGCC7158 encodes type C.2. The ability of the 6073-like EPS-associated phages to adsorb to each of these strains when the 6073-like *eps* gene cluster is present suggests adsorption by the tested phages is independent of the CWPS. This is less clear for the 7127-like *eps* gene cluster, as all three strains tested encode a type A.1 CWPS. However, D4044 and D4351 were unable to adsorb to 7127ΔEPS, indicating that encoding a type A.1 CWPS is not sufficient for adsorption. The P335 phage group is highly diverse (Mahony et al., 2017b), so it is possible that not all members use the CWPS as a receptor. It is unclear if the CWPS is required for subsequent steps in the infection process of these phages that adsorb only to the 6073-like or 7127-like EPS-positive strains, such as DNA injection. The fact that the phages are unable to form plaques on some of the strains they adsorb to shows the infection process is being arrested after the adsorption step, but further study is needed to determine at what point this occurs and whether phage DNA is injected into the cell.

The CWPS structure has been determined for several of the lactococcal CWPS types, revealing an appreciable amount of diversity. While a rhamnose-rich polysaccharide embedded within the peptidoglycan is a conserved feature of the CWPS, the attached oligosaccharide side chains (type A and B) or polysaccharide pellicle (types C and D) are highly variable (Vinogradov et al., 2018a,b; Mahony et al., 2020). It is this variable region that likely encodes the phage receptor, which is believed to explain the narrow host ranges of lactococcal phages (Vinogradov et al., 2018a; Mahony et al., 2020). EPS produced *via* the Wzx/Wzy-dependent pathway also display a high level of structural variability (Zhou et al., 2019). This variability would also be expected to influence phage host range, and this study shows that indeed certain P335 phages require the EPS produced by a specific set of *eps* genes for adsorption. Furthermore, the adsorption of these phages to certain *S. thermophilus* strains indicates the potential for *Lactococcus* and *Streptococcus* to produce EPS with common structures that can be recognized by phages. Despite the commercial relevance of lactococcal EPS, only a small number of structures have been elucidated, revealing diverse heteropolysaccharides, as reviewed elsewhere (Zhou et al., 2019). Determination of the structures of the 6073-like and 7127-like EPS was beyond the scope of this work; however, we believe future studies to elucidate these structures will produce valuable insights regarding phage-host interactions and cell physiology.

While polysaccharides encoded by *rgp* and the *eps* operon have each been identified as receptors for different phages in *S. thermophilus* (Romero et al., 2020), EPS had not previously been associated with phage sensitivity in *Lactococcus.* In fact, whereas our findings demonstrate an association between EPS and sensitivity to phage, previous studies have shown plasmid-encoded lactococcal EPS provides phage resistance. This resistance was characterized as adsorption-inhibition and is theorized to result from the EPS blocking phage cell-surface receptors (Lucey et al., 1992; Forde and Fitzgerald, 1999). While we focus on the association of EPS with P335 sensitivity in this study, we have also found that pEPS6073 provides resistance to certain phages (data not shown). This demonstrates that the role of EPS in phage-host interactions is complex and phage-host specific.

Phage adhesion modules [including distal tail protein (Dit), tail-associated lysin (Tal), and baseplate/receptor-binding protein (Bpp/RBP)] are responsible for attachment to the host (McCabe et al., 2015; Mahony et al., 2017a,b; Hayes et al., 2019). Analysis of the adhesion modules of the phages in this study found them to belong to P335 morphotype II. Dit and Tal show sequence conservation among all phages in this study, but the single-component Bpp/RBP shows sequence conservation within each P335 subgroup. The Bpp/RPB could therefore be used to distinguish the two phage subgroups and to identify additional members of each. Based on this correlation and the established role of phage receptor-binding proteins, the Bpp/RBP is likely responsible for differential adsorption to EPS^+^ strains.

As EPS may be excreted into the growth media or associated with the cell surface, TEM was performed on isogenic EPS^+^/EPS^−^ strain pairs. TEMs of DGCC6073 and DGCC7127 showed uniform fibrillated projections extending from the cell wall. These fibrillated projections are absent from EPS-variants 6073A∆EPS and 7127∆EPS. This indicates that the EPS encoded by pEPS6073 and pEPS7127 are cell surface associated and may be capsular in nature, which is consistent with their roles in phage adsorption.

Phage infection is a leading cause of fermentation failure. An understanding of phage-host interactions is valuable for developing strategies to mitigate phage impact. Identification of host factors involved in phage sensitivity, including EPS, presents targets for strain selection or modification, extending the commercial lifespan of highly specialized starter strains. This study also further extends our understanding of a strain’s spectrum of phage sensitivity and shows strains that do not appear to be phage-related based on plaque formation may still be related by phage adsorption. This has implications for design of commercial cultures which, in most cases, are composed of multiple, phage-unrelated strains. This is meant to increase phage robustness: should phage appear against one strain, the additional phage-unrelated strains can go on to finish the fermentation (Romero et al., 2020). This study indicates that the EPS type of individual strains should be taken into consideration when designing cultures. Furthermore, there may be implications for the design of commercial cultures that are composed of both *Lactococcus* and *S. thermophilus* strains. As the lactococcal P335 phages have already been shown to adsorb to certain *S. thermophilus* strains, it is possible these phages could ultimately evolve to fully infect *S. thermophilus* or recombine with *S. thermophilus* phages to create a new phage type, as was recently described for the streptococcal 987 phage group. 987-group phages possess a P335-like baseplate, encoded by a single gene that shares N-terminal amino acid identity with the P335 BppU (McDonnell et al., 2016). This similarity between the 987 and P335 morphogenesis modules suggests certain phages of both groups may recognize a common host receptor, potentially a common EPS structure, and indeed, representative 987 phages were shown to adsorb to certain *L. lactis* strains in addition to their *S. thermophilus* host (McDonnell et al., 2016). Additionally, analysis of 987-group phage CHPC926 determined that the phage binds to EPS associated with the *S. thermophilus* cell surface (Szymczak et al., 2018). Further study is needed to determine if cultures composed of strains with the same EPS type would be detrimental due to the potential of phages to cross-infect strains or a cost associated with phage adsorption. On the contrary, it is possible that strains that adsorb but do not propagate the phages may effectively remove phage from the environment, “rescuing” fully phage-sensitive strains.

This study identified two related but distinct plasmid-borne *eps* gene clusters in lactococci that we associated with adsorption of certain P335 phages. Although cell-surface polysaccharides are known to be receptors for members of many lactococcal phage groups, including P335, examples to date have been limited to the chromosomally-encoded CWPS. EPS produced *via* the Wzx/Wzy-dependent pathway have been shown to act as phage receptors in other genera, but, to our knowledge, this is the first description in *L. lactis* or *L. cremoris*. Due to the issues caused by phages in the dairy fermentation industry, knowledge of phage-host interactions is critical to developing phage-resistant strains and designing phage-robust starter cultures. This study identified a target for phage resistance and a method to predict potential phage sensitivities based on genome analyses.

## Data availability statement

The data presented in this study have been deposited in the NCBI GenBank repository and can be found at: https://www.ncbi.nlm.nih.gov/genbank/; OP323064-OP323077.

## Author contributions

AM, DR, and LS conceived and designed the experiments. AM performed the experiments. AM, DR, LS, PH, and DM analyzed the data and wrote the paper. AM, PH, and DM performed bioinformatic analyses. AM, DR, PH, and DM contributed reagents/materials/analysis tools. All authors contributed to the article and approved the submitted version.

## Conflict of interest

AM, DR, PH, DM, and LS are all employees of International Flavors and Fragrances (IFF), a company that produces and markets cultures for industrial dairy applications.

## Publisher’s note

All claims expressed in this article are solely those of the authors and do not necessarily represent those of their affiliated organizations, or those of the publisher, the editors and the reviewers. Any product that may be evaluated in this article, or claim that may be made by its manufacturer, is not guaranteed or endorsed by the publisher.

## Footnotes

^1^ https://www.geneious.com
