# Supplementary material for: Host-encoded, cell surface-associated exopolysaccharide required for adsorption and infection by lactococcal P335 phage subtypes
Source: Front Microbiol. 2022 Oct 4;13:971166. doi: 10.3389/fmicb.2022.971166 (PMC9576995; doi:10.3389/fmicb.2022.971166)
Supplement: Supplementary file 1 [file Data_Sheet_1.docx]

Supplementary Material

## Supplementary Figures

**Supplementary Figure 1.** **Additional phage adsorption on representative strains including isogenic strain pairs (+/**- **EPS).** Average of three independent trials. Error bars = sample standard deviation.
