# Supplementary material for: Host-encoded, cell surface-associated exopolysaccharide required for adsorption and infection by lactococcal P335 phage subtypes
Source: Front Microbiol. 2022 Oct 4;13:971166. doi: 10.3389/fmicb.2022.971166 (PMC9576995; doi:10.3389/fmicb.2022.971166)
Supplement: Supplementary file 2 [file Data_Sheet_2.docx]

Supplementary Material

## Supplementary Tables

**Supplementary Table 1: Primer and gblock sequences**

| **Primer name** | **Sequence (5’ – 3’)** |
| --- | --- |
| EpsC-F | CTAGTCCGACTATAACAGATATC |
| EpsC-R | GAAGGAAACACAGGAACAGAC |
| 6073 gblock-F | ATGAATAAAATAACCATGACAAG |
| 6073 gblock-R | AGCCTCTTTCTGATCCATTATTAC |
| check 6073 SNP-F | AGAACTTCAGGATATATCTG |
| check 6073 SNP-R | GTGGTAAAACTGTAATGCTC |
| 6073 revert-F | AGAACTTCAGGATATATCTT |
| 7127 gblock-F | TAATAAGAAAAGTAACAGTTATATTTTTGC |
| 7127 gblock-R | CCTTCTCCTGTCTCTCATTC |
| check 7127 SNP-F | CCTTTATTTTCTTTAATACCCAATG |
| check 7127 SNP-R | GGTCGATGACTTATACTATCC |
| 7127 revert-F | CCTTTATTTTCTTTAATACCCAAAA |
| 6073 gblock | ATGAATAAAATAACCATGACAAGAGAGATGAGAGTGGTTGCCTTATGTGTCGTAATTTTAGAATATTTAAATAATACAGGATTAATTGCGTCTTCAGTATACTCTTTTAGCATGGCGAGTACAATCCTCTTATCCTATATCTTATTCTGTAAAAAAAGAGAAGGATTTTCTTTAAAGGAGATTATTGTACTACTAATTTCCTTTATTTTTGTAGTTTTAAATCGTAATGCTAGTAATTTTAGTTTAGGGTTAATGTGTATACTCTATTTTATGTTAAGTAAGTCGGAAATAGATTTAAAAAAAGTGATGAAAACATTTTTTGTTACCTCTAGTGTTTGTTTTATTTTGACAATATTACTTTATTTAATAATGTCTCTTAATAAAAGCTCTGATATGATAATGTGGCGTGGAGATGCTTTTATCAATCGTATGAGTTTAGGATTTATCCAACCGAATTTTGCAATGATAAGCTTTTTAGGTGTAGCGATAGCGTTATTATATTTGAGTACTGAAAGACAAAGAATAACTATAATTTTTATTGCCATTTTAACTTTTATTATATTTTACTTTACTCAATCAAGAACTTCAGGATATATCTGATTTTTTATTTTGAGTATTTTATTTGTTAGTAGTAAAAAAACTAAAAAGCAAGTTTCAAATTTTGAAAAATGGAGCATTACAGTTTTACCACTAATTCTTTTAATAATCTCTTATTCGTTGTTAAAGTTACCTATTAATCAACACCTCAATAACTTGCTTTCTGGTCGTCTGTCGCTTTATCAAGAGATTTATTCTACATTTGGTATACATTTGATAGGGAATAATGATGTTAAAAATACAATGTTAGATTCAGCATATCTTCAAAGTTTGCTAGCAAAAGGAATTTTGTTTACATTGTTTTTATTTGTAACTTTCTTTTTCATATTTTTTCTTAAGAGAAAAACACAAACTAGGTTGCAAAGTTTAGTAATTATGATGTATTTTTTAATTGCATTTACAGAAACATCATTTTTTAGGTTTGTAATTTTATTTCCAGTATTGATGGTAATAATGGATCAGAAAGAGGCT |
| 7127 gblock | GTAGTTTTAATTGGTATTATCTTATACTTAGGAGGCTATCCTCTAAACAAATTTTTGGGAATAGATGAAATTTACTACGCTAAGGGGGTAACTACTTTACTTAATAATTTTGATGGCAGATTTGGATCAGATGTATTTGGAATTTCCGTAACGCGTATGGGATCAATTTATTTTGAACCAATAAATTTGGGGTATTTAATATTTTCTATGCTCATTATATCCTTTATTTTCTTTAATACCCAAAAATTAAAATATATAAATTTATATAGACTGATTTTATTGATAGGTGGAATGTTAACTTTTGGGAAAGGTGCTATGCTTCTAGCTATAGGGGTGATGGTTGCAGGTATCGGACATAAATTATTTTTAAAATTCTTTCCAAGAAGTAATGAAATGAATGTTTTTAGAAATCTTTTTATATTGTTAACTATTATTATGTTTATTGGTGGAAATTATTATTTCAAAACTTTTGGAGGAGCTGTAGGGAATCACTTTTATGCAATCCAAGGGACATTGGATAGTATAAGTCATCGACCAATTGGTTTTGGATTAGGTGTGGGAGGAAACGCCTCAGCAGTATTTACAGGGGGAGAACTTGATTTTACTACTGGATCAGAAACAGCCTTATTATCATTTGTATACCAAATAGGTGTTCAAGGTGCTATTGCTTTAATATGTATATTCTACTTTATGAGTAAAGAAGTATTGGAAAAAGTACAAAAGAATTCACAATTCAAAAATAGATTTTTATTTTATGTTCCAATGATATTGATTTTTGTTAGTATATATCAAGCTAATACATATACCCCACAATGTATAACGTTGTTAATGATTACTTTGGGAGGATTTGTAGGAATGAGAGACAGGAGAAGG |

**Supplementary Table 2: Natural competence PCRs**

| **Strain** | **Transformation Amplicon** | | **Screening primers** |
| --- | --- | --- | --- |
|  | primers | template |  |
| DGCC12520 | 6073 gblock-F; 6073 gblock-R | 6073 gblock | check 6073 SNP-F; check 6073 SNP-R |
| 12520ΔEPS | 6073 gblock-F; 6073 gblock-R | DGCC12520 | 6073 revert-F; check 6073 SNP-R |
| DGCC7856 | 7127 gblock-F; 7217 gblock-R | 7127 gblock | check 7127 SNP-F; check 7127 SNP-R |
| 7856ΔEPS | 7127 gblock-F; 7127 gblock-R | DGCC7856 | 7127 revert-F; check 7127 SNP-R |

## Supplementary Figures

**Supplementary Figure 1.** **Additional phage adsorption on representative strains including isogenic strain pairs (+/**- **EPS).** Average of three independent trials. Error bars = sample standard deviation.
